# Facial Micro-Expression Recognition Using Double-Stream 3D Convolutional Neural Network with Domain Adaptation

**DOI:** 10.3390/s23073577

**Published:** 2023-03-29

**Authors:** Zhengdao Li, Yupei Zhang, Hanwen Xing, Kwok-Leung Chan

**Affiliations:** 1 Department of Electrical Engineering, City University of Hong Kong, Hong Kong, China; zhengdali3@gmail.com (Z.L.); hanwexing2-c@my.cityu.edu.hk (H.X.); 2 Centre for Intelligent Multidimensional Data Analysis Limited, Hong Kong, China; ypzhang5-c@my.cityu.edu.hk

**Keywords:** micro-expression recognition, 3D-CNN, optical flow, domain adaptation

## Abstract

Humans show micro-expressions (MEs) under some circumstances. MEs are a display of emotions that a human wants to conceal. The recognition of MEs has been applied in various fields. However, automatic ME recognition remains a challenging problem due to two major obstacles. As MEs are typically of short duration and low intensity, it is hard to extract discriminative features from ME videos. Moreover, it is tedious to collect ME data. Existing ME datasets usually contain insufficient video samples. In this paper, we propose a deep learning model, double-stream 3D convolutional neural network (DS-3DCNN), for recognizing MEs captured in video. The recognition framework contains two streams of 3D-CNN. The first extracts spatiotemporal features from the raw ME videos. The second extracts variations of the facial motions within the spatiotemporal domain. To facilitate feature extraction, the subtle motion embedded in a ME is amplified. To address the insufficient ME data, a macro-expression dataset is employed to expand the training sample size. Supervised domain adaptation is adopted in model training in order to bridge the difference between ME and macro-expression datasets. The DS-3DCNN model is evaluated on two publicly available ME datasets. The results show that the model outperforms various state-of-the-art models; in particular, the model outperformed the best model presented in MEGC2019 by more than 6%.

## 1. Introduction

Facial expressions are one of the natural means for humans to convey feelings and intentions. Automatic facial expression recognition (FER) has been an active research topic in the past decades due to its potential applications in various fields such as psychology and human–computer interaction. For successful recognition, it is important to extract effective facial features. For instance, Shan et al. [1] derived discriminative facial representation from two-dimensional (2D) images based on the local binary pattern (LBP) descriptor. Features extracted from facial appearance images are sensitive to illumination and pose variations. To address these problems, facial representation has also been derived from geometrical information [2]. Fan and Tjahjadi developed spatiotemporal frameworks for FER that introduced the integration of three-dimensional (3D) facial features and dense optical flow [3], a spatiotemporal feature based on the Zernike moment, and a dynamic feature comprising motion history image and entropy [4].

In addition to ordinary facial expressions, also called macro-expressions, humans may show micro-expressions (MEs) under some circumstances. MEs are often genuine emotions that a human wants to conceal for some reason. For instance, to deceive others in order to gain an advantage or avoid loss. Applications of ME recognition (MER), e.g., in police interrogation, assessment of patients’ psychological states, etc., can have significant social impact. Unlike macro-expressions, MEs are subtle and often imperceptible. Thus, it is difficult to detect and recognize MEs. In fact, the recognition accuracy achieved by humans without training is slightly better than chance [5]. Even with time-consuming training, the performance of experts is less than satisfactory. Recently, automatic MER has attracted research interest [6]. It is a challenging problem as ME is characterized by its short duration and comprises local facial movements with low intensity. While some FER algorithms, e.g., [7], may achieve accuracy over 90%, the performance of recent MER systems is mostly in the range of 60–70%. Some publicly available ME databases were created to facilitate MER research. The SAMM dataset [8] comprises 133 samples—92 others, 26 happiness, and 15 surprise. The SMIC dataset [9] comprises 164 samples—70 negative, 51 positive, and 43 surprise. The samples were recorded by a high-speed camera, visual camera, and near-infrared camera. The CASME II database [10] includes 247 samples which were recorded at 200 fps. Li et al. [11] proposed an ME analysis system with two major steps: ME spotting and ME recognition. ME spotting is used to detect the onset, apex, and offset of MEs. The apex occurs when the change in facial muscle reaches the peak or the highest intensity of the facial motion. Therefore, the apex frame is the instant indicating the most expressive emotional state in a video. The features being used are LBP and the histogram of oriented gradients. For recognition, methods are introduced to amplify the motion and lengthen the duration of ME. The ME classification is performed by a linear support vector machine (SVM). Huang et al. [12] enhanced the image features of ME by computing the LBP from three orthogonal planes (LBP-TOP). Mid-level features are learned from low-level features extracted from facial regions in [13].

Recently, computer vision has advanced rapidly through the use of deep learning. In contrast to deterministic algorithms, deep learning is machine learning based on learning data representations. It has led to advances in FER and MER, e.g., [14]. Khor et al. [15] proposed a recurrent deep network for MER. The framework uses the convolutional neural network (CNN) to encode every single ME frame of a video into a characterized vector and uses long short-term memory (LSTM) to predict the ME classes. Li et al. [16] proposed a 3D flow-based CNN model to study MER in videos. The model extracts the tiny facial movements caused by MEs. Zhou et al. [6] presented a comprehensive survey on datasets and MER algorithms that are grouped according to the feature extraction approach (i.e., handcrafted or deep learning).

In this paper, we propose a deep learning model, double-stream 3D convolutional neural network (DS-3DCNN), for recognizing MEs in video. Our main contributions are as follows:

- While most MER research only inputs the apex frame, or a few key frames, of the ME video, we propose a framework for MER with an image sequence as the input. This type of input ensures that more spatiotemporal information is provided for the subsequent analysis process. In constructing the image sequence, we make sure that the apex frame is included. Thus, the image sequence is not only very simple to generate but also preserves essential facial information.
- The recognition framework comprises two streams of 3D-CNN for extracting representative features from the photometric and motion information. The first extracts spatiotemporal features from the raw ME image sequence. The second extracts variations of the optical flow vectors within the spatiotemporal domain. The two sets of features extracted by the two streams are fused and further analyzed for facial expression prediction. As demonstrated in our high-prediction accuracy in MER, the multi-stream framework is very effective in extracting subtle facial motion in MEs.
- In order to provide more training video samples, we adopted supervised domain adaptation. Most MER research with domain adaptation uses training and testing MEs from two different ME datasets. However, there are few ME datasets and they contain a small number of samples. On the other hand, we observe that there are more macro-expression datasets with a large number of samples. Thus, we propose our model which learns not only from training samples from ME datasets but also from training samples from macro-expression datasets.
- Experimentations were performed to optimize the major hyperparameters of the proposed MER framework. As a result, our model still extracts facial motion features better, even though the facial motion is subtle, and the datasets contain irrelevant variations such as background and head posture. The experimental results show that our framework achieves higher accuracy than the baseline model and the other models presented in the 2019 Facial Micro-Expressions Grand Challenge.

The organization of this paper is as follows. The related studies are reviewed in Section 2. We focus on various deep learning models proposed for MER. Section 3 describes our proposed double-stream framework and the pre-processing of the ME videos. Moreover, we explain the concept of supervised domain adaptation, which is adopted in order to provide more training samples. We evaluate our proposed framework and compare its performance with various state-of-the-art models. Section 4 presents the experimental results and comparative analyses. Section 5 presents the ablation studies to demonstrate the optimality of each major part of our proposed framework. Finally, we conclude our work in Section 6.

## 2. Related Work

With the development of CNN [17] and graphics processing units (GPUs), significant advancement in computer vision has been reported. Researchers have found that learned features can vastly outperform traditional algorithms by training the deep learning model with a large dataset. There are many relevant CNN models for FER and MER. Fan et al. [18] directly transferred the successful visual geometry group (VGG) [19] structure to the FER task, with deep supervision in each layer. Wang et al. [20] applied the common attention mechanism of image classification to MER and achieved improvement in accuracy. Khor et al. [21] and Liu et al. [22] employed optical flow information, estimated from onset and apex frames, as inputs to CNN.

One main difficulty of MER research is the lack of ME datasets. To address this limitation, Lopes et al. [23] proposed some image pre-processing steps to extract specific features of ME. The recognition is performed by a deep CNN. Alternatively, Wang et al. [24] resorted to transfer learning and proposed the transferring long-term CNN model. Takalkar et al. [25] proposed a framework to extract and integrate handcrafted features (LBP-TOP) and deep CNN features.

The above-mentioned algorithms focus on classification based on a single or a few RGB images. Although the approach reduces computation costs, it does not fully exploit the underlying motion and temporal information in the video. To address this, Zhao et al. [26] constructed a key frame sequence from the onset, apex, and offset frames. Optical flow estimated from the key frame sequence is input to 3D-CNN. Ji et al. [27] proposed 3D-CNN, which is able to extract spatiotemporal features between consecutive image frames for action recognition. Haddad et al. [28] utilized 3D convolution in FER and achieved desirable results. Recently, Reddy et al. [29] utilized such 3D convolution in MER. One of the shortcomings of 3D-CNN is its expensive computation. Indeed, there are redundant parameters in 3D convolution and thus the model can easily become overfitted to small datasets. Reddy et al. [29] built a shallow yet powerful 3D-CNN to avoid this problem. They also proposed another model with the input of sub-regions (e.g., eyes, mouth) cropped from the face images. However, the performance is worse than the model that uses whole face images.

In addition to 3D-CNN, a two-stream structure is also popular in motion-related/temporal-related events recognition. Specifically, Simonyan et al. [30] input RGB images and their corresponding optical flows to two parallel 2D CNNs. By doing this, the first stream (i.e., spatial stream) with RGB input extracts spatial features, while the second stream (i.e., temporal stream) with optical flow input extracts motion features. Since there is no 3D convolution in this structure, the computation cost is significantly reduced. Following this idea, Khor et al. [21] proposed a shallow dual-stream CNN for MER. The authors built a dual-stream shallow network (DSSN), which significantly outperformed the baseline method without optical flow input.

Video data are essentially a sequence of continuous images. Consequently, one can also utilize a recursive neural network (RNN) which is proven to be robust when modeling sequential data such as for machine translation and voice recognition. Peng et al. [31] extracted spatial features from the apex frames using CNN and temporal features with the LSTM network. Khor et al. [15] first computed the optical flow of each frame and built the RNN in a two-stream structure. However, two drawbacks in RNN-like structures (e.g., vanilla RNN, LSTM, and gated recurrent unit (GRU)) are: (1) When the parameters in RNNs are updated, the gradients are computed by backpropagation through time (BPTT). If the sequences are long, then the gradients that backpropagate to the earlier stages are prone to disappear or explode. (2) Unlike speech data in natural language processing, image data are far more complex, containing noise and irrelevant features. RNN-like structures are sometimes not robust enough to process image data.

The accuracy of the deep learning model is substantially affected by the amount of training samples. Thus, in practice, deep learning algorithms require sufficient datasets. On the other hand, MEs are spontaneous and only occur in a blink. Hence, most of the ME datasets are relatively small. Therefore, data augmentation is often employed to extend the training set. Augmentation methods such as translation, rotation, and flipping are commonly used to generate synthetic data. For instance, horizontal flipping of the facial images was used in [25] to double the training set. Xia [32] proposed two temporal data augmentation methods to overcome the problem of imbalanced training samples. The first extends the training set based on the multiple-scale amplification factors. In the second method, data samples are randomly selected for augmentation. With both methods applied jointly, the training set can be extended by fifty times. Takalkar et al. [33] proposed utilizing data augmentation techniques to enlarge datasets, leading to better recognition performance. Yu et al. utilized a generative adversarial network (GAN) [34] to enlarge the size of training samples by generating synthetic MEs.

Most research in MER utilizes training and testing samples from the same dataset. In this case, the training and testing samples share the same feature distribution. In a practical application, the MER system will face the challenge of a large feature distribution difference between the training and testing samples. For instance, the training and testing MEs are from two different datasets. Therefore, the expressions are likely to be captured under different environments (e.g., different illumination) and by different equipment (e.g., different types of camera or different frame rates). Thus, the performance of MER will deteriorate. To address this problem, cross-database ME recognition has been proposed [35,36]. The training and testing samples are from two different ME datasets: CASME II [10] and SMIC [9]. A classifier such as SVM learns from the labeled ME samples. Thus, in testing, the classifier predicts the labels of the un-labeled ME samples. Zhang et al. [37] proposed a regression network for MER learned from multiple datasets. They compared six domain adaptation methods on three datasets CASME II, SAMM, and SMIC. Song et al. [38] proposed a dual-stream CNN to address the problem of using labeled ME source samples and unlabeled ME target samples. Liu et al. [39] adopted transfer learning that can select facial regions contributing to features for distinguishing different MEs.

## 3. Proposed Model

**Figure 1:**
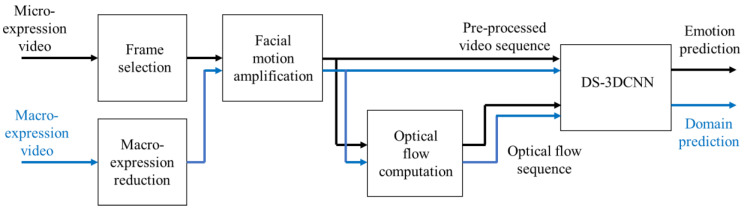
Overview of our proposed MER framework.

We first elaborate on the proposed MER framework and the features extracted by the two streams of 3D-CNN. Then, we describe the pre-processing steps to prepare the inputs for 3D-CNN. Finally, we describe the techniques, such as domain adaptation, which are used to enrich the training video samples and enhance the learned model. Figure 1 shows the block diagram with all the major stages of our proposed MER framework. Details of these stages are explained in the following sections.

### 3.1. Proposed Double-Stream 3D-CNN

**Figure 2:**
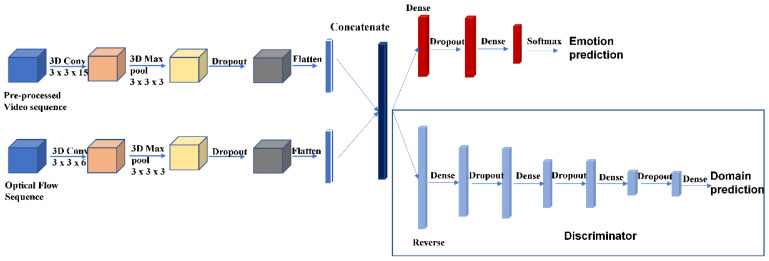
Structure of the proposed DS-3DCNN.

The proposed model, DS-3DCNN, is composed of two individual input streams that converge in the middle of the model. The overall structure of the model is shown in Figure 2. The pre-processed video sequence is input to the first stream (video stream). The 3D convolution operation is performed with 32 filters, followed by a 3 × 3 × 3 max pooling and dropout. The kernel size of 3 × 3 is good for the extraction of fine details in images. This size is commonly adopted in many deep learning models for image analysis. The filter dimension and the number of channels follow the convolutional layer structure in some 3D-CNN such as [29]. Finally, the feature vectors are flattened into a 1-dimensional (1D) array before the convergent step. Table 1 shows the network components of the video stream.

The second stream (optical flow stream) has a similar structure as the first but with the optical flow sequence as input. The 3D convolution operation is performed with 32 filters of 3 × 3 × 6. Another difference is that the strides of the convolution are set as (1, 1, 2) instead of (1, 1, 1). This is because each optical flow is computed from two layers in the temporal axis and the optical flow is considered as one inseparable unit. Table 2 shows the network components of the optical flow stream.

The convergence of the model is achieved by concatenating the two 1D arrays from the two streams. The result is a 1D array with both features extracted from the video sequence and the optical flow sequence. The array is processed with two different sub-structures. The first sub-structure consists of two dense layers, one dropout layer and one softmax layer, to produce the prediction for the emotion label. For both the SAMM and SMIC datasets, there are three emotion labels, namely, negative, positive, and surprise. Therefore, the output dimension of the softmax layer is 1 × 3. The second sub-structure is the discriminator which consists of a reverse layer, three dense layers, three dropout layers, and the last 1 × 1 dense layer (which is a softmax layer). The dropout value in all the dropout layers, similar to VGG [19], is set to 0.5. The gradient reversal layer (GRL) is a layer that multiplies a constant in backpropagation. In forward propagation, it just passes the value forward.

In order to achieve higher accuracy in MER, the framework should have the input of complete spatiotemporal information. Therefore, we utilize the whole face region in the video sequence. Moreover, motion information is generated from the original image sequence rather than from a few key frames. The dense optical flow sequence has the same resolution in the temporal domain as the video sequence. With the two sequences as inputs, we therefore adopt the multi-stream structure for the proposed framework. Each stream is a 3D-CNN, which is trained efficiently and is also very effective in feature extraction. The first input sequence embeds the raw ME video in a 3D data cube. The 3D-CNN is trained to extract spatiotemporal features from the pre-processed video sequence. Traditional CNNs may only have one or a few RGB images input into the network. They are not effective to extract temporal features. On the contrary, 3D-CNNs with the input of the image sequence are more capable of extracting representative features from both spatial and temporal dimensions of the motion data. The second 3D-CNN, with the input of the optical flow sequence, extracts variations of the facial motion. The feature extracted by this network shares a similar concept as the flux tensor representation. The flux tensor corresponds to the temporal variation of the optical flow field within the 3D spatiotemporal domain [40]. It has been successfully applied in various video understanding systems such as moving object detection [41]. Here, representative features are extracted with the use of 3D-CNN. The network is sensitive to optical flow gradients and is thus trained to extract features of subtle facial motion. The two streams of 3D-CNN provide high-diversity feature vectors which guarantee high MER accuracy. We adopted the ensemble solution to fuse the two feature vectors at an intermediate location of the framework. This design ensures that more layers are provided for further analysis of the concatenated feature.

### 3.2. Pre-Processing

**Figure 3:**
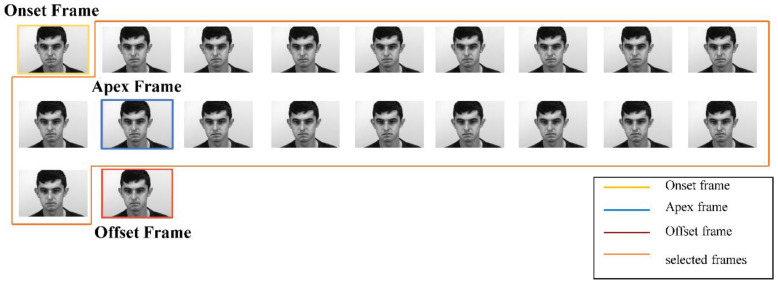
Frame selection with 9 frames before the apex frame and 8 frames after the apex frame.

**Figure 4:**
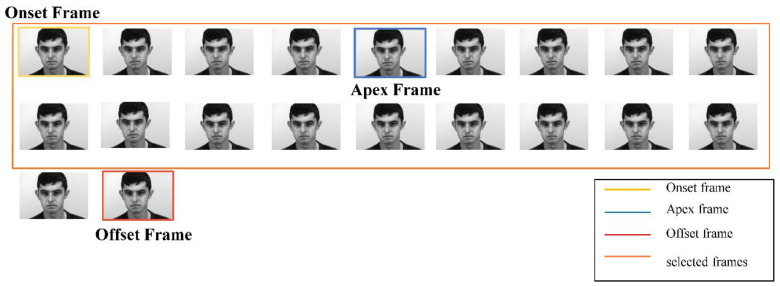
Frame selection with less than 9 frames before the apex frame.

The ME videos may contain complications that are unrelated to micro-expressions. For instance, there may be a change in the head posture, e.g., due to rotation. Therefore, with the aid of detected facial landmarks, the face region is aligned by normalizing the orientation of the face. The original video sequence is transformed into two different 3D matrices. These matrices will be input into the DS-3DCNN model. The first 3D matrix is the resized video sequence. This sequence is generated in two steps. The first step is frame selection, where 18 frames are selected from the video sequence enclosing the apex frame of the ME as illustrated in Figure 3. For the SAMM dataset, where there are fewer than 9 frames before the apex frame, the first 18 frames of the sample are selected as illustrated in Figure 4. Similarly, when there are fewer than 9 frames after the apex frame, the last 18 frames of the sample are selected. For the SMIC dataset, the first 18 frames of each video are selected since the apex frame is not denoted in the dataset. For videos that do not have 18 frames, the offset frame is duplicated and selected until there are 18 frames in total.

**Figure 5:**
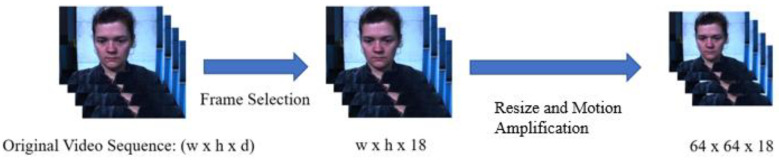
Generation of the processed video sequence.

The second step involves resizing and concatenating. Each image frame is resized into 64 × 64 by interpolation and the resized frame is processed with a motion magnification algorithm which is described below. Finally, image frames are concatenated in the temporal axis resulting in a 64 × 64 × 18 matrix (i.e., width × height × depth) for 3D convolution. The generation of the processed video sequence is illustrated in Figure 5.

**Figure 6:**
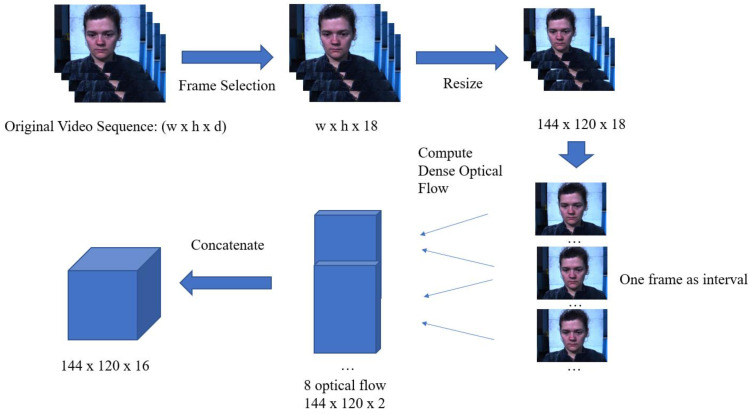
Generation of the optical flow sequence.

The second 3D matrix is the optical flow sequence which is generated by a three-step procedure. The first step is similar to that of the first 3D matrix. Following frame selection, the image in the video sequence is resized into a 144 × 120 matrix using the same interpolation method. The dimension for the optical flow sequence is set based on two considerations: the image size of the dataset and the motion magnification algorithm. Firstly, we have to set the dimension to be the smallest image size of all datasets in order to allow our proposed models to fit to all datasets. Secondly, the motion magnification algorithm performs upscaling and downscaling of the image. To be able to do these, the dimension of the sequence also needs to be adjusted to be divisible by a certain value. The matrix dimension of 144 × 120 is the largest that meets these two requirements. The dense optical flow between two image frames with an interval of d is then computed using the Gunnar Farneback method [42]. For example, when d is 2, frame 0 and frame 2 generate one dense optical flow field. Similarly, frame 2 and frame 4 generate the next dense optical flow field, and so forth. In total, 8 dense optical flow fields, each with the size of 144 × 120 × 2, computed from the 18 selected video frames are generated. Finally, all dense optical flow matrices are concatenated in the temporal axis. This results in a 144 × 120 × 16 3D matrix, the optical flow sequence, and is input to the second stream of DS-3DCNN. The generation of the optical flow sequence is illustrated in Figure 6.

**Figure 7:**
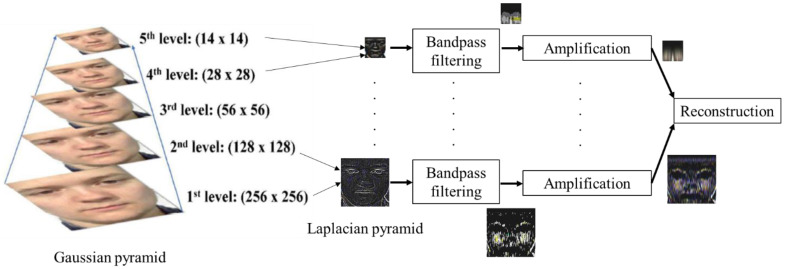
The flow of pre-processing of the ME video by EVM.

**Figure 8:**
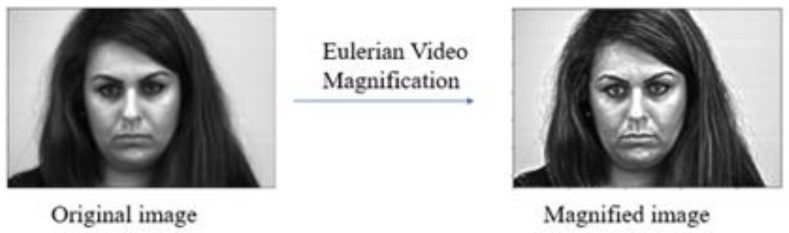
Amplification of facial motion by EVM.

In order to improve MER accuracy, facial motion magnification is performed. The motion amplification algorithm adopted is the Eulerian video magnification (EVM) [43] technique as it amplifies subtle motions in video and has been applied widely in various ME recognition tasks, e.g., [32]. The algorithm decomposes a video input into a number of spatial frequency bands *L* in the form of a Laplacian pyramid as shown in Figure 7. Each spatial band is temporally processed with the bandpass filter which preserves facial motion and attenuates other frequencies. The filter output *B* is amplified by a factor *α* and added back to the original spatial band input, i.e., *L′* = *L* + *αB*. Finally, the pyramid *L′* is collapsed and the resulting video is reconstructed with the motion magnified. From our ablation study as shown in Section 5, the optimal value of *α* is 20. Figure 8 illustrates the effect of EVM when applied to one ME image.

### 3.3. Enhanced Model Learning

**Figure 9:**
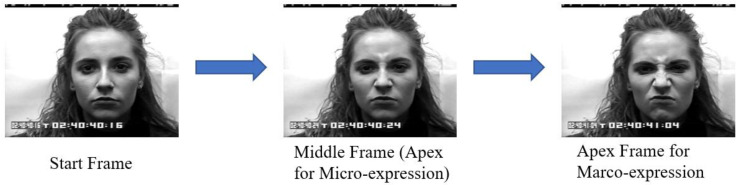
The start frame, middle frame, and apex frame of a sample in the CK+ dataset. The middle frame is selected as the apex frame of the training image sequence.

More training videos are needed to enhance the learned model. Therefore, the macro-expression dataset CK+ [44,45] is included in the training dataset. Due to the limited number of image frames in some CK+ samples, a few frames are duplicated in order to meet the requirement of 18 frames for the 3D data input. Macro-expression may be different from micro-expression. Thus, in order to use the macro-expression dataset for training our MER model, it is necessary to maximize the similarity between macro-expression and micro-expression. Therefore, we adopted the macro-expression reduction [22]. The algorithm assumes that the apex of ME is very similar to the intermediate expression between the onset and apex of macro-expression. Based on this idea, the middle frame of a macro-expression is selected as the apex frame of the image sequence to be used for model training as illustrated in Figure 9.

**Figure 10:**
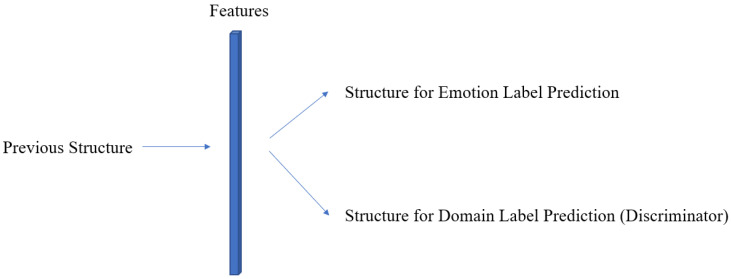
Features from a previous structure are processed both by the structures for emotion prediction and the discriminator.

Furthermore, in order to accommodate the macro-expression dataset for model training, we adopted the supervised domain adaptation technique [46]. To bridge the difference between a macro-expression dataset and a ME dataset, the proposed framework incorporates a discriminator that differentiates the video samples from different datasets. The discriminator is implemented in the model as a branch that processes features generated from the previous structure of the model as illustrated in Figure 10.

The discriminator changes the loss function for optimization, introducing the gap between the samples from different datasets. Thus, the loss function *L* after adopting the supervised domain adaptation technique is

(1) $$L=\sum_{i=0}^{i=N}L_{y}^{i}\left(y_{i}\left(\theta_{f},\theta_{y},x_{i}\right),y_{i}^{\prime }\right)-\lambda L_{d}^{i}\left(d_{i}\left(\theta_{f},\theta_{d},x_{i}\right),d_{i}^{\prime }\right)$$

where *θ_f_* is the trainable parameter in a previous structure and *θ_d_* is the trainable parameter in the discriminator, while *θ_y_* is the trainable parameter for the network that predicts the emotion label. The parameters *y_i_*(*θ_f_*, *θ_y_*, *x_i_*) and *d_i_*(*θ_f_*, *θ_d_*, *x_i_*) are respectively the predicted values of emotion label and domain label for sample *x_i_*, while *y_i_′* and *d_i_′* are respectively the true emotion and domain label values for sample *x_i_*. *L_y_*(*y_i_*, *y_i_′*) is the loss function for predicting the emotion label, i.e.,

(2) $$L_{y}^{i}=-\sum_{c=1}^{M}y_{i}^{\prime }\times \log\left(y_{i}\right)$$

where *M* represents the total number of emotion classes and *y_i_𠌩* and *y_i_* represent the true label and predicted label of emotion, respectively. *L_d_*(*d_i_*, *d_i_′*) is the loss function for predicting the domain, i.e.,

(3) $$L_{d}^{i}=-[d_{i}^{\prime }\times \log\left(d_{i}\right)+(1-d_{i}^{\prime })\times \log\left({1-d}_{i}\right)]$$

where *d_i_′* and *d_i_* denote the true label and predicted label of the domain, respectively. The total number of training samples is *N*. The hyperparameter *λ* is introduced to control the influence of domain adaptation by assigning weight to the loss function of the domain prediction.

## 4. Experiments

The performance of the proposed DS-3DCNN model is evaluated on two ME datasets: SAMM [8] and SMIC [9]. Labels for both datasets are classified into three categories. The SAMM dataset comprises 133 samples—92 other, 26 happiness, and 15 surprise. The SMIC dataset comprises 164 samples—70 negative, 51 positive, and 43 surprise. SMIC contains different types of samples. We used the samples captured by a high-speed camera with the cropped face region images provided in the SMIC dataset. The SAMM dataset does not have cropped face images. Therefore, we used the full-face images in our experiments. We did not perform face cropping. The macro-expression dataset CK+ [44,45] contains 327 samples, which are also classified into three categories.

We adopted the leave-one-subject-out cross-validation (LOSOCV) method to ensure that the evaluation was independent of the validation subject. In LOSOCV, the features of the sample videos in one subject are treated as the testing data, and the remaining features from the rest of the subjects become the training data. The SAMM dataset has 28 participants. Therefore, the training and validation process is performed 28 times. Similarly, the SMIC dataset has 16 participants, and the training and validation process is performed 16 times. To prevent biases due to limited sample size and disproportional distribution of labels, unweighted F1-score (*UF*1) and unweighted Average Recall (*UAR*) were adopted as evaluation metrics, e.g., in MEGC2019 [47]. True positive (*TP_c_*), false positive (*FP_c_*), and false negative (*FN_c_*) are counted for each class *c*. The total number of classes is *C*. Thus,

(4) $$UF1=\sum_{i}^{C}\frac{{UF1}_{i}}{C}$$

where

(5) $${UF1}_{c}=\frac{2\times {TP}_{c}}{2\times {TP}_{c}+{FP}_{c}+{FN}_{c}}$$

In addition,

(6) $$UAR=\frac{1}{C}\sum_{i}^{C}{Acc}_{i}$$

where

(7) $${Acc}_{c}=\frac{{TP}_{c}}{n_{c}}$$

and *n_c_* denotes the total number of video samples in class *c*. Some published works presented the result in terms of mean accuracy, which is the ratio of the number of true positives to the total number of samples.

The model is trained on a computer with AMD EPYC 7742 64 Cores × 2 CPU, HPE DL385 × 4 GPU, and 512 GB memory. Table 3 shows the model complexity and run-time analysis of our proposed models and other models. DS-3DCNN without domain adaptation has a total number of model parameters of 16,660,963. The computation load is 517 MFLOPs and the execution time per subject is 25.63 s. DS-3DCNN with domain adaptation has a total number of 33,325,796 model parameters. The computation load is 550 MFLOPs and the execution time per subject is 91.65 s. The loss function for emotion is the categorical cross entropy loss function and the loss function for the domain is set as the binary cross entropy loss function (i.e., Equation (1)). The model is trained with the stochastic gradient descent (SGD) optimization technique [48] using the default learning rate of 0.01. Each training includes running 200 epochs with a batch size of 8.

In the first experiment, we compared the result of our two proposed models with the baseline model LBP-TOP [52] and the models published in MEGC2019 as shown in Table 4. As shown in the second last row of Table 4, DS-3DCNN without domain adaptation achieved a substantial improvement in comparison with the baseline model LBP-TOP [52]. Our model also outperformed the three models proposed in [51,53,54] in MEGC2019. The impact of image size can be seen by comparing our proposed models with [51]. We resized the images to 64 × 64, while Liong et al. [51] resized the images to 28 × 28. Our proposed models outperformed [51] in all evaluation metrics on both datasets. Table 4 also shows the significance of domain adaptation. As shown in the last row of Table 4, the adoption of domain adaptation leads to an improvement of up to 5.6%. The three evaluation metrics exceeded the highest results provided in MEGC2019 using the model proposed in [22] by up to 6.8%. Overall, DS-3DCNN without domain adaptation mostly achieved the second-highest scores. While with domain adaptation, our model achieved the highest *UAR* score on both the SAMM and SMIC datasets and the highest *UF*1 score on the SMIC dataset. Liu et al. [22] presented 2 sets of results, with and without adversarial domain adaptation. Our model, DS-3DCNN, without domain adaptation outperformed Liu’s model without adversarial domain adaptation on both datasets and on all evaluation metrics. Our model, DS-3DCNN, with domain adaptation had a UF1 score on the SAMM dataset only slightly lower than Liu’s model with adversarial domain adaptation. This is because we used full-face images from SAMM, while Liu et al. [22] used part-based (eyes and mouth) feature extractors. Therefore, our results could be improved with cropped faces.

Other published works presented the result in terms of mean accuracy. Therefore, in the second experiment, we compare our proposed models with other models based on this metric. Zhao et al. [26] utilized an optical flow sequence synthesized from three key frames and a single 3D-CNN. The framework by Sun et al. [55] incorporates a fusion of features extracted from the apex frame and optical flow estimated from two key frames. Table 5 shows the mean accuracy of DS-3DCNN without domain adaptation, DS-3DCNN with domain adaptation, and other models on the SAMM dataset. This comparative analysis demonstrates the significance of the utilization of dense motion information. The proposed model DS-3DCNN with domain adaptation outperforms the other methods by more than 4%.

Reddy et al. [29] proposed two models: the first has the input of the whole face image sequence fed to a single 3D-CNN and the second has the inputs of eyes and mouth regions fed to two streams of 3D-CNN. Table 6 shows the mean accuracy of DS-3DCNN without domain adaptation, DS-3DCNN with domain adaptation, and other models on SMIC dataset. This comparative analysis demonstrates the advantages of the use of whole-face images and the double-stream framework. The proposed model DS-3DCNN with domain adaptation outperforms the other methods by more than 10%.

**Figure 11:**
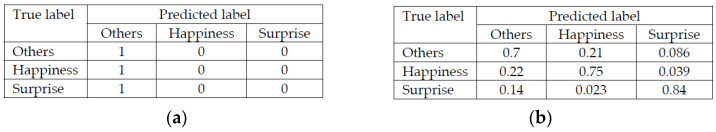
Confusion matrices of DS-3DCNN without domain adaptation: (**a**) SAMM dataset and (**b**) SMIC dataset.

**Figure 12:**
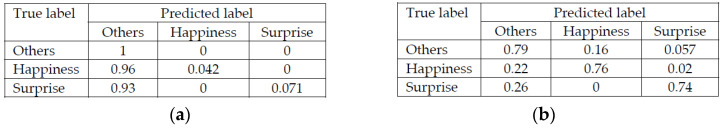
Confusion matrices of DS-3DCNN with domain adaptation: (**a**) SAMM dataset and (**b**) SMIC dataset.

To demonstrate more detailed ME classification results, the confusion matrices of DS-3DCNN without domain adaptation and DS-3DCNN with domain adaptation are shown in Figure 11 and Figure 12, respectively. In each matrix, the diagonal values correspond to the truly predicted expressions. Generally, DS-3DCNN with domain adaptation performs better than DS-3DCNN without domain adaptation in both datasets. Our model recognizes other/negative emotions with high accuracy. On the SAMM dataset, the misclassification of happiness and surprise emotions are affected by the imbalanced sample distribution. On the SMIC dataset, the distribution of labels is more even, and our model achieves higher accuracies compared with other works such as Reddy et al. [29] and Sun et al. [55].

**Figure 13:**
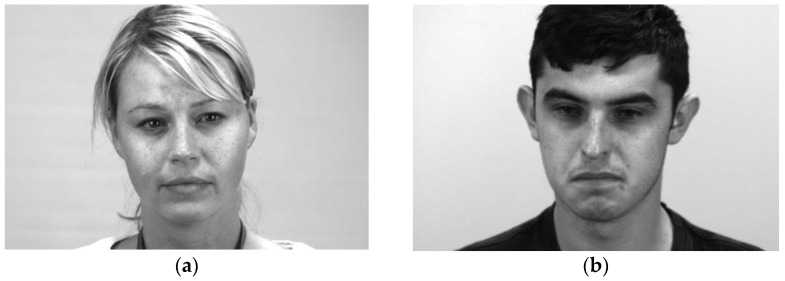
Correct recognition results on the SAMM dataset: (**a**) happy and (**b**) other.

**Figure 14:**
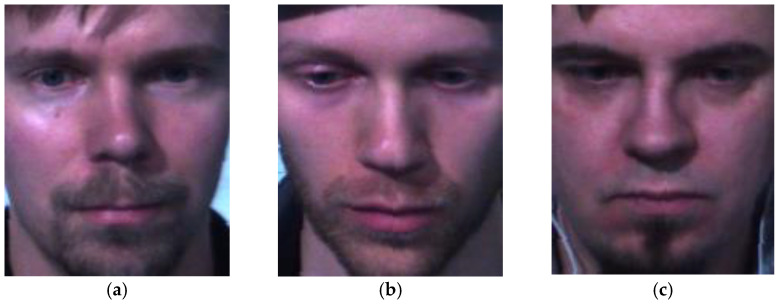
Correct recognition results on the SMIC dataset: (**a**) positive, (**b**) negative, and (**c**) surprise.

**Figure 15:**
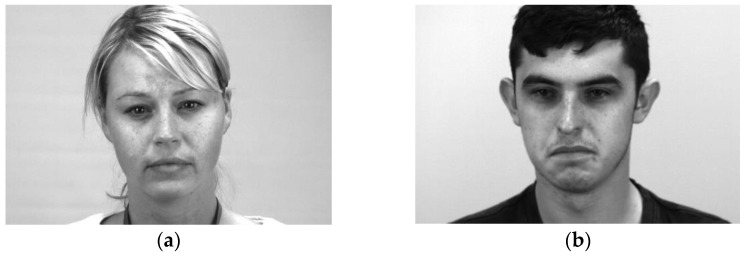
Incorrect recognition results on the SAMM dataset: (**a**) happy predicted as other and (**b**) surprise predicted as other.

**Figure 16:**
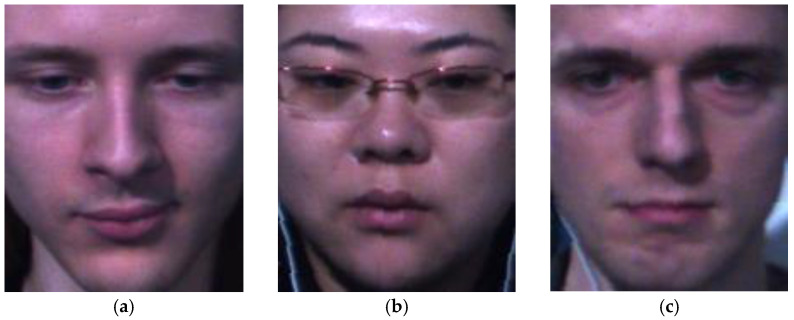
Incorrect recognition results on the SMIC dataset: (**a**) positive predicted as negative, (**b**) negative predicted as surprise, and (**c**) surprise predicted as negative.

Figure 13 and Figure 14 show some correct recognition results on the SAMM dataset and SMIC dataset, respectively. Figure 15 shows some incorrect recognition results on the SAMM dataset. Comparing Figure 13a and Figure 15a, there is a subtle change at the corners of the mouth that resulted in misclassification. The incorrect recognition of Figure 15b is obvious as it looks very similar to Figure 13b. Figure 16 shows some incorrect recognition results on the SMIC dataset. Again, emotion may be misclassified as another type that is visually similar. Fortunately, the model trained on the SMIC dataset has very good performance and there were only a few misclassification cases.

## 5. Ablation Studies

We performed ablation studies on EVM and the loss function. Some hyperparameters were selected for experimentation. First, we compared the performance of DS-3DCNN with or without using EVM. Table 7 shows the results of DS-3DCNN without using EVM on the two datasets. Without domain adaptation, utilizing EVM to amplify the facial motion leads to a slight improvement in accuracy (e.g., 1.6% on SAMM). With domain adaptation, the effect of EVM is more significant. Improvement is seen in all evaluation metrics. Utilizing EVM leads to a 4% improvement in SAMM and a 5% improvement in SMIC.

Next, we investigated the effect of the amplification factor in EVM on the DS-3DCNN model’s performance. Table 8 shows the results of DS-3DCNN without domain adaptation on the two datasets with different amplification factors. Table 9 shows the results of DS-3DCNN with domain adaptation on the two datasets with different amplification factors. Since the face motion of MEs is very subtle, small amplification cannot facilitate the extraction of useful features. On the other hand, if the amplification is too large, the performance of the model will deteriorate due to image distortion and noise. The results show that the best choice for the amplification factor is 20.

Finally, we investigated the effect of the hyperparameter *λ* in the loss function (Equation (1)) on the DS-3DCNN model’s performance. Table 10 shows the results of the two datasets with different *λ*. The hyperparameter regulates the contribution of the discriminator in model training. If it is small, the macro-expression samples will not affect the optimization of the model parameters. If it is too large, the model will be trained to better recognize macro-expression instead of micro-expression. The results show that the best choice for *λ* is 15.

## 6. Conclusions

We propose a deep learning model, DS-3DCNN, for recognizing MEs captured in a video. MEs are subtle and often imperceptible. Thus, to address this challenge, we propose the framework which contains two streams of 3D-CNN. The first stream extracts the spatiotemporal feature from the photometric data of the original image sequence. The optical flow vectors are fed to the second stream of 3D-CNN, which is trained to detect the changes in motion. Most of the ME datasets are relatively small. Thus, to address these difficulties, our model adopts the supervised domain adaptation. In so doing, the macro-expression dataset, which comprises more samples, can be used for model training. A series of experiments are performed to optimize the proposed model. The evaluation of the DS-3DCNN model on two ME datasets, SMIC and SAMM, shows that our proposed model outperforms various state-of-the-art models.

Although the results of our work show that our model is superior to others, there are still ways to improve it. In this study we chose 3D convolutions for feature extraction. In a complex network, 3D convolutions contain a large amount of parameters and require high computation costs. In the future, we will investigate a computationally efficient framework for MER. For instance, we will investigate temporal modeling in video sequences using a pooling technique to achieve fewer parameters, thus requiring less computation.

## Figures and Tables

**Table 1:** Network components of the video stream.

Layers	# Channels	Filter Dimension	Output Size
Video sequence	-	-	64 × 64 × 18
Convolution	32	3 × 3 × 15	32 × 62 × 62 × 4
Max pooling	-	3 × 3 × 3	32 × 20 × 20 × 1
Dropout	-	-	32 × 20 × 20 × 1
Flatten	-	-	12,800

**Table 2:** Network components of the optical flow stream.

Layers	# Channels	Filter Dimension	Output Size
Optical flow sequence	-	-	144 × 120 × 16
Convolution	32	3 × 3 × 6	32 × 142 × 118 × 6
Max pooling	-	3 × 3 × 3	32 × 47 × 39 ×2
Dropout	-	-	32 × 47 × 39 × 2
Flatten	-	-	117,312

**Table 3:** Comparison of model complexity and run-time analysis between the proposed models and state-of-the-art models.

Method	# Parameters	FLOPs	Execution Time (s)
Bi-WOOF [49]	-	-	128.7134
OFF-ApexNet [50]	1.3 M	-	-
Liong et al. [51]	0.002 M	-	5.7366
Khor et al. [21]	0.97 M	-	-
DS-3DCNN without domain adaptation	16 M	517 M	25.63
DS-3DCNN with domain adaptation	33 M	550 M	91.65

**Table 4:** Comparison of *UAR* and *UF*1 scores between two proposed models (DS-3DCNN without domain adaptation and DS-3DCNN with domain adaptation) and state-of-the-art models. The best results are highlighted in red and the second-best results are highlighted in blue.

Method	SAMM		SMIC	
	*UAR*	*UF*1	*UAR*	*UF*1
LBP-TOP [52]	0.4102	0.3954	0.5280	0.2000
Bi-WOOF [49]	0.5139	0.5211	0.5829	0.5727
OFF-ApexNet [50]	0.5392	0.5409	0.6695	0.6817
Quang et al. [53]	0.5989	0.6209	0.5877	0.5820
Zhou et al. [54]	0.5663	0.5868	0.6726	0.6645
Liong et al. [51]	0.6810	0.6588	0.7013	0.6801
Liu et al. [22]	0.7152	**0.7754**	**0.7530**	0.7461
**DS-3DCNN without domain adaptation**	**0.7425**	**0.7564**	0.7500	**0.7611**
**DS-3DCNN with domain adaptation**	**0.7830**	0.7554	**0.8061**	**0.7887**

**Table 5:** Comparison of mean accuracy between two proposed models and other models on the SAMM dataset.

	SAMM (Mean Accuracy)
Zhao et al. [26]	0.6403
Sun et al. [55]	0.7500
Khor et al. [21]	0.5735
OFF-ApexNet [50]	0.6818
**DS-3DCNN without domain adaptation**	**0.7825**
**DS-3DCNN with domain adaptation**	**0.7917**

**Table 6:** Comparison of mean accuracy between two proposed models and other models on the SMIC dataset.

	SMIC (Mean Accuracy)
Reddy et al. [29] (one 3D-CNN)	0.6875
Reddy et al. [29] (two-stream 3D-CNN)	0.6482
Sun et al. [55]	0.6585
Khor et al. [21]	0.6341
OFF-ApexNet [50]	0.6768
**DS-3DCNN without domain adaptation**	**0.7692**
**DS-3DCNN with domain adaptation**	**0.7878**

**Table 7:** Performance of DS-3DCNN with and without using EVM.

Model	SAMM		SMIC	
	*UAR*	*UF*1	*UAR*	*UF*1
DS-3DCNN (without EVM, without domain adaptation)	0.7283	0.7402	0.7790	0.7607
DS-3DCNN (without EVM, with domain adaptation)	0.7462	0.7535	0.7827	0.7377
DS-3DCNN (with EVM, without domain adaptation)	0.7425	0.7564	0.7500	0.7611
DS-3DCNN (with EVM, with domain adaptation)	0.7830	0.7554	0.8061	0.7887

**Table 8:** Performance of DS-3DCNN (without domain adaptation) with different amplification factors in EVM.

Amplification Factor	SAMM		SMIC	
	*UAR*	*UF*1	*UAR*	*UF*1
10	0.6992	0.7156	0.7635	0.7214
15	0.7371	0.7449	0.7277	0.6723
20	**0.7425**	**0.7564**	0.7500	**0.7611**
25	**0.7425**	**0.7564**	**0.7753**	0.7258
35	0.7143	0.7267	0.7634	0.7445

**Table 9:** Performance of DS-3DCNN (with domain adaptation) with different amplification factors in EVM.

Amplification Factor	SAMM		SMIC	
	*UAR*	*UF*1	*UAR*	*UF*1
10	0.7234	0.7428	0.7590	0.7036
15	0.7371	0.7449	0.7486	0.6925
20	**0.7830**	**0.7554**	**0.8061**	**0.7887**
25	0.7371	0.7430	0.7150	0.6624
35	0.6992	0.7156	0.7769	0.7400

**Table 10:** Performance of DS-3DCNN (with domain adaptation) with different *λ* in the loss function.

*λ*	SAMM (Mean Accuracy)	SMIC (Mean Accuracy)
1	0.7825	0.7410
5	0.7899	0.7536
10	0.7825	0.7690
15	**0.7917**	**0.7878**
20	0.7862	0.7606

## Data Availability

Not applicable.
